# Anterior segment structures in dark iris Chinese patients with unilateral Fuchs’ uveitis syndrome

**DOI:** 10.1007/s10792-022-02282-w

**Published:** 2022-04-02

**Authors:** Yajing Cai, Wenjie Wu, Yingbin Wang, Suzhen Xiao, Yue Huang

**Affiliations:** 1 Provincial Clinical Medical College, Fujian Medical University, Fuzhou, Fujian Province China; 2 Department of Ophthalmology, Fujian Provincial Hospital, 134, Dongjie Rd, Fuzhou, 350001 Fujian Province China

**Keywords:** Fuchs, Uveitis syndrome, Anterior segment structure, Dark iris, Chinese patients

## Abstract

**Purpose:**

To compare binocular anterior segment structures in Chinese patients with dark iris and unilateral Fuchs’ uveitis syndrome (FUS).

**Methods:**

This was a cross-sectional study including 34 phakic eyes (17 patients) with unilateral FUS. Anterior segment parameters were measured by rotating Scheimpflug imaging camera, noncontact specular microscopy, and anterior segment optical coherence tomography.

**Results:**

Corneal volume was higher in FUS eyes compared to unaffected eyes (*p* < 0.05). The iridocorneal angles were larger in FUS eyes compared to contralateral eyes (*p* < 0.05). Mean endothelial cell density (ECD) was lower, and the coefficient of variation in endothelial cell size and average cell area of endothelial cells (ACA) were higher, in FUS eyes (*p* < 0.05). Mean densitometry values of the midstromal cornea (zones with a diameter of 0–2, 2–6, or 10–12 mm), posterior (0–2, 2–6, 10–12, or 0–12 mm), or total thickness (0–2 or 2–6 mm) were higher in FUS eyes compared with unaffected eyes (*p* < 0.05). ECD, percentage of hexagonal cells, and ACA were strongly related to densitometry values of the midstromal and posterior cornea in the FUS eyes (*p* < 0.05). Smoothness index of iris was lager in affected eyes (*p* < 0.05).

**Conclusion:**

In Chinese patients with unilateral FUS, loss of endothelial cells, wider iridocorneal angle, thicker cornea, higher corneal densitometry of midstromal and posterior layer, and smoother iris were observed in affected eyes compared to contralateral eyes. These data can help to elucidate anterior segment characteristics of unilateral FUS in this population.

## Introduction

Fuchs' uveitis syndrome (FUS) is a chronic, typically unilateral, mild inflammatory disorder, which predominantly involves the anterior uvea [1]. It was first described by Ernst Fuchs in 1906 [2]. In the world, FUS accounts for 1–20% of all anterior uveitis cases [1]. Characterized by fine stellate keratic precipitates (KPs), mild anterior chamber reaction, iris atrophic changes with or without heterochromia, vitreous involvement, and absence of macular edema or posterior synechiae [3–5], it affects both genders equally, occurring most frequently in the third and fourth decades of life [5–8]. It is also worth noting that heterochromia was more prominent in white patients with light-colored eyes compared to those with dark or brown eyes [6]. As diagnosis is primarily based on clinical features versus laboratory examination, FUS is frequently incorrectly diagnosed, especially in patients with dark irises [9, 10]. In China, where most population appears dark brown iris, FUS accounts for 7% of all uveitis patients, but only 7.7% patients referred to the uveitis center were correctly diagnosed by the primary ophthalmologist [6]. Many studies have concentrated on identifying changes in anterior segment structures in eyes with FUS and have shown promising results contributing to accurate diagnosis [6, 11–18]. Currently, using advanced instruments, further details of the anterior segment disorders of FUS eyes are elucidated. Using Scheimpflug imaging, researchers identified corneal and anterior chamber characteristics in FUS eyes including corneal thickness, corneal densitometry values, iridocorneal angle, and iris bowing [12, 14]. Using anterior segment optical coherence tomography (AS-OCT), Zarei et al. [11] and Invernizzi et al. [19] described iris morphology alterations in FUS eyes by a “smoothness index” and iris thickness. Further, investigators have analyzed endothelial changes in eyes with FUS by specular microscope [12, 14, 16, 18]. Simsek et al. [14] demonstrated the relationship between corneal densitometry and endothelial cell function. However, to our knowledge, these anterior segment biometric parameters of FUS eyes in dark iris Chinese patients have not been reported. Yang et al. [6] reported on clinical features of Chinese patients with FUS by using auxiliary examinations including laser flare-cell photometry, ultrasound biomicroscopy (UBM), fundus fluorescein angiography (FFA), and serologic tests for Toxoplasma gondii. Yet, because Scheimpflug imaging, AS-OCT, and noncontact specular microscopy were not applied, subtle anterior segment biometric characteristics in this population were insufficient. To supplement the Chinese data, we measured corneal, iris, and anterior chamber parameters in unilateral FUS eyes by means of currently available advanced ophthalmic instruments.

## Patients and methods

This cross-sectional comparative study was performed at Fujian Provincial Hospital between March 2020 and May 2021. The study followed the tenets of the Declaration of Helsinki and approval from the Institutional Ethics Board was obtained.

Thirty-four phakic eyes among 17 consecutive Chinese patients with unilateral FUS were enrolled. The irises of all unaffected eyes were dark brown, which was the most common iris color among Asians [20]. Diagnosis was made by one experienced ophthalmologist based primarily on clinical features, including stellate or round small- to medium-sized KPs, chronic anterior uveitis, iris atrophy with or without heterochromia, absence of posterior synechiae, or absence of macular edema. Posterior subcapsular cataract, vitreous opacity, and secondary glaucoma are supportive for FUS, but are not essential for the diagnosis.

FUS-like cases, such as Posner–Schlossman syndrome (PSS), HSV/ VZV anterior uveitis, and cytomegalovirus endotheliitis, were excluded. Among them, acute and recurrent attacks, no heterochromia, and positive responds to steroids suggested PSS. Hypertensive granulomatous uveitis, reduced corneal sensation, corneal scars, neurotrophic ulcers, and sectoral iris atrophy from previous episodes were considered to be HSV/ VZV anterior uveitis, especially along with skin lesions. Corneal endothelitis with KPs arranged in a coin-shaped pattern strongly indicated cytomegalovirus endotheliitis. Meanwhile, patients with corneal abnormalities (opacities), intumescent cataract, a history of bilateral uveitis, bilateral FUS, any intraocular surgery, ocular trauma, or using medication that affects pupillary diameter were excluded from the study. Patients with lighter iris color than dark brown were not included under the consideration that they belong to very minority population in China. Patients with previous elevated intraocular pressure (IOP) well controlled with anti-glaucomatous agents were included.

A comprehensive ophthalmic examination was performed in both eyes of all patients including best-corrected visual acuity (BCVA), slit-lamp biomicroscopy, IOP, and fundus examination. Corneal endothelial parameters were measured using Topcon Specular Microscope SP-3000 (Topcon Corporation, Tokyo, Japan). Anterior segment imaging was performed on each eye using a rotating Scheimpflug camera (Pentacam HR Oculus Optikgera¨te GmbH, Germany) and AS-OCT scanning (Cirrus HD-OCT Model 5000, Carl Zeiss Meditec, Inc). All patients were examined in a sitting position by the same examiner who was masked to their eye disease status. Anterior segment images were obtained under the same brightness condition, e.g., Scheimpflug camera in dim room lighting and AS-OCT in regular day room illumination.

Corneal and anterior segment biometric parameters were measured by Scheimpflug imaging including central corneal thickness (CCT), corneal volume, anterior chamber depth, anterior chamber volume, anterior chamber angles, undilated pupil size, anterior and posterior keratometric values (anterior K1, K2 and posterior K1, K2, respectively), and corneal densitometry. Corneal densitometry was analyzed by densitometry software, which automatically divided the cornea into four concentric zones (with diameters of 2 mm, 2–6 mm, 6–10 mm, or 10–12 mm) and into three layers (120 μm beneath the epithelium, 60 μm above the endothelium, and the remaining middle layer). Only when being labeled as “OK” by the Examination Quality Indicator of the Pentacam were the images included and analyzed.

**Fig. 1:**
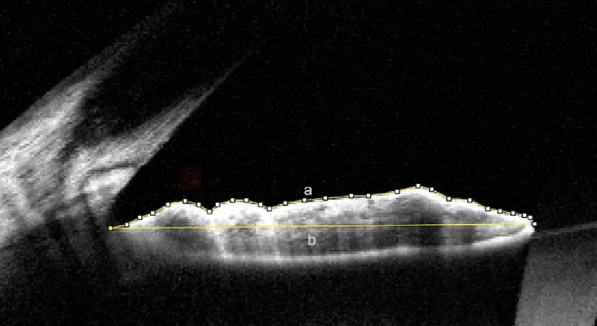
Smoothness Index (SI); ratio of the length of the straight line connecting the most peripheral and the most central points of anterior iris surface (**b**) to actual length of this boundary (**a**). Overall SI is the sum length of nasal and temporal “straight lines” divided by the sum of nasal and temporal actual lengths of anterior iris boundary

Images acquired from AS-OCT were used to calculate the iris smoothness index (SI). The iris was scanned horizontally on 3 and 9 o’clock position. If the examiner noted any artifact or images were blurred during scanning, the measurement was repeated until an acceptable image was achieved. The selected images were exported to and analyzed with ImageJ (ImageJ version 1.52, NIH, USA) software by one author who was masked to the affected eye. The Segmented Line tool and Strait Line tool of ImageJ were used to measure lengths with 300% magnification. SI was the ratio of length of the straight line connecting the most peripheral and the most central points of the anterior iris surface (nasal and temporal sides) to the actual length of this boundary (nasal and temporal sides) (Fig. 1) [11]. The overall SI was defined as the sum of length of nasal and temporal “straight lines” divided by the sum of nasal and temporal actual lengths of the anterior iris boundary [11].

### Statistical analysis

Analyses were conducted using SPSS (IBM SPSS Statistics for Windows, Version 26.0. Armonk, NY). To describe data, we used mean, standard deviation, range, and percentage. Variables were investigated using the Shapiro–Wilk test to determine whether they were normally distributed. Comparisons were between two eyes of patients with unilateral FUS wherein the unaffected eye served as the control; therefore, paired sample *t*-test and Wilcoxon signed-rank test analysis were used. Correlation coefficients’ significance was calculated with the Pearson test. A *p*-value < 0.05 was considered statistically significant.

## Results

### Demographic and clinical features

This study included a total of 34 eyes among 17 participants (11 males, 6 females; aged 25–67 years) with unilateral FUS. The mean age was 46.82 ± 12.34 years. Heterochromia was seen in 9 subjects. Posterior subcapsular cataract and glaucoma, respectively, were found in 2 (11.8%) and 15 (88.2%) FUS eyes. One subject with subcapsular cataract underwent uncomplicated phacoemulsification cataract surgery one week after study entry, during which Amsler–Verrey sign was observed. Previously, 15 affected eyes were treated with anti-glaucoma eye drops (beta blocker and/or carbonic anhydrase inhibitor) and short-term steroids were used if necessary (Table 1).

**Table 1:** Demographic and clinical features of unilateral FUS patients

	Mean ± SD (range)	Percentage (%)
Age (years)	46.8 ± 12.34 (25–67)	
Gender (M/F)	11/6	65/36
Laterality (right/left)	7/10	41/59
BCVA (logMAR)		
FUS	0.22 ± 0.49	
Unaffected eye	0.06 ± 0.22	
Anterior segment signs		
KPs	17/17	100
Cell/flare aqueous	3/17	18
Iris depigmentation	9/17	53
Amsler–Verrey sign	1/17	6
Posterior subcapsular cataract	2/17	12
Vitreous opacity	3/17	18
IOP (mmHg)		
FUS eye	16.8 ± 3.3	
Unaffected eye	17.4 ± 2.7	

*SD* Standard deviation; *F* Female; *M* Male; *BCVA* Best-corrected visual acuity

### Corneal and anterior segment biometric parameters

Central corneal thickness, corneal volume, and anterior and posterior keratometric values were recorded and compared between FUS eyes and unaffected eyes. The corneal volume was larger in FUS eyes than in unaffected eyes (59.60 ± 3.42 mm^3^ and 58.84 ± 3.20 mm^3^, respectively; *p* = 0.005). Anterior chamber depth and volume, undilated pupil size, and iridocorneal angles were also compared. The average iridocorneal angles was wider in FUS eyes than in contralateral eyes (38.10 ± 5.41° and 36.01 ± 4.77°, respectively; *p* = 0.007) (Table 2).

**Table 2:** Comparison of the anterior segment parameters between FUS eyes and contralateral eyes

	FUS eye	Healthy eye	*p* Value
Central corneal thickness (μm)	538.80 ± 37.01	537.69 ± 35.84	0.787
Anterior keratometric value (K-1)	43.25 ± 1.24	43.13 ± 1.22	0.198
Anterior keratometric value (K-2)	43.95 ± 1.43	43.92 ± 1.38	0.768
Posterior keratometric value (K-1)	− 6.16 ± 0.21	− 6.14 ± 0.21	0.105
Posterior keratometric value (K-2)	− 6.41 ± 0.18	− 6.39 ± 0.20	0.198
Corneal volume (mm^3^)	59.60 ± 3.42	58.84 ± 3.20	0.005^*^
Anterior chamber volume (mm^3^)	162.13 ± 39.55	162.46 ± 37.09	0.927
Anterior chamber depth (mm)	2.89 ± 0.33	2.88 ± 0.32	0.565
Undilated pupil size (mm)	2.78 ± 0.43	2.60 ± 0.51	0.045^*^
Mean anterior chamber angle (°)	38.10 ± 5.41	36.01 ± 4.77	0.007^*^

*Statistically significant

The mean densitometry values of the center (zones with a diameter of 0–2 mm, 2–6 mm, or 10–12 mm), posterior (0–2 mm, 2–6 mm, 10–12 mm, or 0–12 mm), and total thickness (0–2 mm or 2–6 mm) were significantly higher in FUS eyes compared with unaffected eyes (*p* < 0.05). The mean densitometry values of the remaining zones showed no significant differences (Table 3).

**Table 3:** Corneal densitometry measurements of the patients with unilateral FUS

	FUS eye	Healthy eye	*p* Value
*Anterior (mm) (GSU)*			
0–2	25.30 ± 4.77	24.02 ± 2.61	0.076
2–6	23.01 ± 3.52	21.95 ± 2.32	0.066
6–10	26.47 ± 6.88	25.49 ± 5.56	0.326
10–12	41.08 ± 10.59	37.23 ± 8.93	0.150
Total (0–12)	27.32 ± 4.64	25.92 ± 3.29	0.123
*Center (mm) (GSU)*			
0–2	16.18 ± 5.44	14.50 ± 0.85	0.012^*^
2–6	14.16 ± 2.06	13.34 ± 0.89	0.022^*^
6–10	18.02 ± 4.75	17.36 ± 4.08	0.423
10–12	25.04 ± 4.25	22.37 ± 3.77	0.034^*^
Total (0–12)	17.51 ± 2.84	16.33 ± 1.78	0.066
*Posterior (mm) (GSU)*			
0–2	15.52 ± 3.71	14.02 ± 0.93	0.002^*^
2–6	13.99 ± 1.75	13.13 ± 1.00	0.008^*^
6–10	17.55 ± 3.39	16.93 ± 3.10	0.073
10–12	21.78 ± 3.27	20.25 ± 1.94	0.044^*^
Total (0–12)	16.67 ± 2.28	15.67 ± 1.54	0.018^*^
*Total thickness (mm) (GSU)*			
0–2	19.00 ± 4.53	17.50 ± 1.25	0.001^*^
2–6	17.06 ± 2.38	16.14 ± 1.22	0.007^*^
6–10	20.68 ± 4.87	19.92 ± 4.10	0.381
10–12	29.29 ± 5.54	26.62 ± 4.61	0.067
Total (0–12)	20.51 ± 3.15	19.30 ± 2.07	0.058

GSU, grayscale unit, is the pixel luminance per unit volume in the Scheimpflug image

*Statistically significant

### Corneal endothelial cell parameters

Mean endothelial cell density, mean values of coefficient of variation in cell size (polymegathism or CV), average cell area (ACA), and percentage of hexagonal cells (pleomorphism or HEX) were compared between FUS eyes and unaffected eyes. (Table 4). There were significantly strong correlations between densitometry values of some zones in the midstromal, posterior, and total corneal layer ECD, HEX, and ACA (*p* < 0.05 and r > 0.50 for the majority of these comparisons) (Table 5).

**Table 4:** Endothelial cell characteristics of the patients with unilateral FUS

	FUS eye	Healthy eye	*p* Value
ECD (cell/mm^2^)	2320.2 ± 329.0	2596.9 ± 240.6	0.001^*^
CV (%)	35.4 ± 6.8	30.6 ± 3.5	0.004^*^
HEX (%)	56.6 ± 9.9	60.2 ± 10.5	0.258
ACA (μm^2^)	441.5 ± 81.8	388.3 ± 36.9	0.002^*^

*ECD* Endothelial cell density; *CV* (polymegathism), coefficient of variation in cell size; *HEX* (pleomorphism), percentage of hexagonal cells; *ACA* Average cell area

*Statistically significant

**Table 5:** Correlation between the specular microscopy measurements and corneal densitometry values in affected eyes of the patients with FUS

	ECD	HEX	ACA	CV
*Center (mm)*				
0–2	*P* = 0.001^*^	*P* = 0.023^*^	*P* < 0.001^*^	*P* = 0.487
	r = − 0.716	r = 0.546	r = 0.862	
2–6	*P* = 0.003^*^	*P* = 0.012^*^	*P* < 0.001^*^	*P* = 0.519
	r = − 0.677	r = 0.592	r = 0.796	
6–10	*P* = 0.318	*P* = 0.239	*P* = 0.392	*P* = 0.491
10–12	*P* = 0.338	*P* = 0.797	*P* = 0.318	*P* = 0.837
Total (0–12)	*P* = 0.011^*^	*P* = 0.040^*^	*P* = 0.004^*^	*P* = 0.993
	r = − 0.600	r = 0.501	r = 0.657	
*Posterior (mm)*				
0–2	*P* = 0.003^*^	*P* = 0.045^*^	*P* < 0.001^*^	*P* = 0.631
	r = − 0.672	r = 0.492	r = 0.822	
2–6	*P* = 0.009^*^	*P* = 0.066	*P* = 0.001^*^	*P* = 0.819
	r = − 0.612		r = 0.736	
6–10	*P* = 0.346	*P* = 0.682	*P* = 0.374	*P* = 0.244
10–12	*P* = 0.186	*P* = 0.799	*P* = 0.131	*P* = 0.913
Total (0–12)	*P* = 0.030^*^	*P* = 0.244	*P* = 0.011^*^	*P* = 0.759
	r = − 0.526		r = 0.601	
*Total thickness (mm)*				
0–2	*P* = 0.003^*^	*P* = 0.020^*^	*P* < 0.001^*^	*P* = 0.484
	r = − 0.683	r = 0.560	r = 0.832	
2–6	*P* = 0.007^*^	*P* = 0.013^*^	*P* < 0.001^*^	*P* = 0.521
	r = − 0.628	r = 0.586	r = 0.754	
6–10	*P* = 0.241	*P* = 0.344	*P* = 0.255	*P* = 0.578
10–12	*P* = 0.352	*P* = 0.912	*P* = 0.382	*P* = 0.911
Total (0–12)	*P* = 0.024^*^	*P* = 0.051	*P* = 0.011^*^	*P* = 0.999
	r = − 0.542		r = 0.602	

*Statistically significant

*ECD* Endothelial cell density; *CV* (polymegathism), coefficient of variation in cell size; *HEX* (pleomorphism), percentage of hexagonal cells; *ACA* Average cell area

### Smoothness index

The mean SI values of temporal, nasal, and total iris, respectively, were higher in FUS eyes (0.909 ± 0.061, 0.924 ± 0.026, and 0.916 ± 0.040) compared to contralateral eyes (0.876 ± 0.063, 0.897 ± 0.056, and 0.885 ± 0.054). These differences were statistically significant (*p* = 0.035, *p* = 0.044, and *p* = 0.004).

## Discussion

Demographic and clinical features of FUS in different ethnic groups have been widely reported [6, 10]. Many manifestations of FUS patients like age, gender, bilateral involvement, mild inflammation in the anterior chamber (characteristic KPs, minimal cells and flare in the aqueous), iris nodules, absence of posterior synechiae, vitreous opacities, cataract, and glaucoma were described similarly between Chinese and other races [6, 10]. However, heterochromia was less common in Chinese patients in contrast to less pigmented patients, while was consistent with heavily pigmented ones [6]. Since more subtle changes were difficult to detect directly by the human eye even with careful slit-lamp examination, in this study, we primarily applied advanced imaging techniques including Scheimpflug imaging and AS-OCT in dark iris Chinese patients with unilateral FUS to analyze the anterior segment morphology changes. These data can help to elucidate anterior segment characteristics of unilateral FUS in this population.

Previous studies described changes in CCT in FUS eyes and drew different conclusions. Szepessy et al. [12] found significantly thinner CCT in FUS eyes using Scheimpflug imaging with a 20-μm difference between affected and unaffected eyes. Conversely, using the same imaging technology, Simsek et al. [14] reported that CCT was comparable between both eyes of unilateral FUS cases. The studies of Basarir et al. [13] (measured with ultrasonographic pachimetry) and Goker et al. [21] (device not known) also detected similar results. Consistently, there was no statistically significant difference in CCT between FUS eyes and unaffected eyes in our study. However, these studies did not compare corneal volume. Corneal volume may be a more meaningful measure than CCT to detect change in corneal thickness and to monitor corneal swelling [22–25]. What is more, according to Takács, Suzuki and co-investigators, change in corneal volume due to swelling after phacoemulsification persisted longer than that of CCT [22, 23]. In our research, we found that corneal volume, centered on the vertex with a diameter of 10 mm, was significantly larger in FUS eyes. This result indicates that there was invisible corneal swelling in affected eyes even though the cornea was “all clear” by slit-lamp examination. Given that a large proportion of our subjects had secondary glaucoma, we speculate that micro-edema of the cornea might be due to repeatedly elevated IOP.

**Fig. 2:**
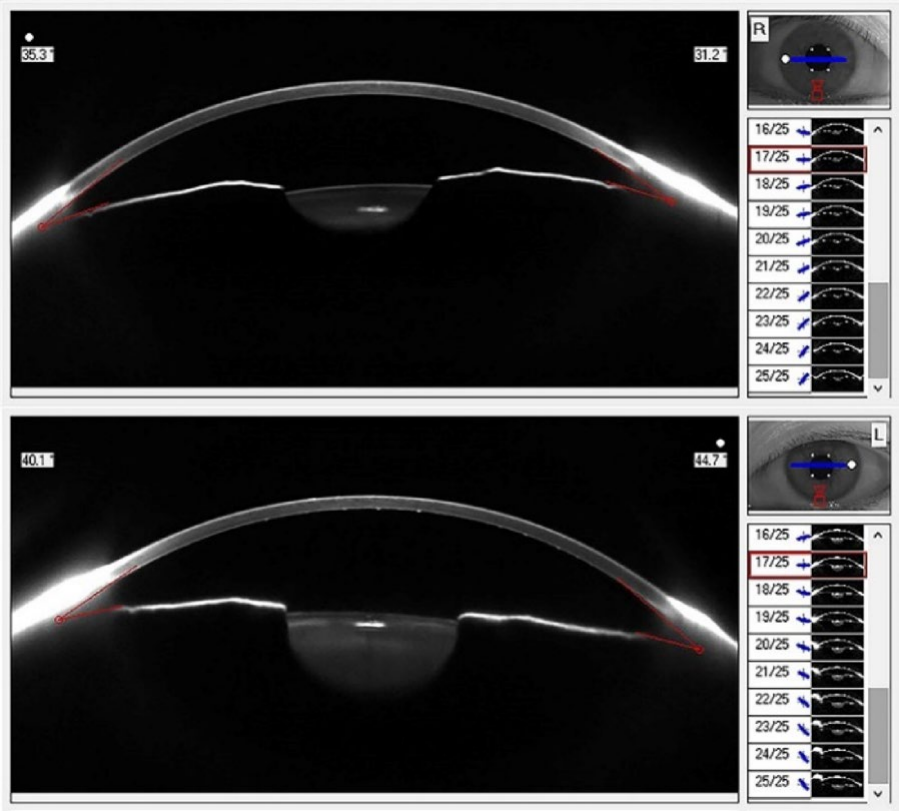
Anterior segment image of a unilateral FUS patient. The many hyper-reflective points correspond to KPs on the inner surface of the cornea in the affected (left) eye. At the top is the unaffected eye and at the bottom is the FUS eye

The corneal densitometry obtained through light backscattering measurements provide precise information about corneal transparency [26]. The density is expressed in grayscale unit (GSU) ranged from 0 to 100. Zero means no opacification of cornea (maximum transparency) and 100 means completely opaque cornea (no transparency). In our study, significantly increased corneal densitometry was observed in FUS eyes in midstromal zones (0–2, 2–6, or 10–12 mm), posterior zones (0–2, 2–6, 10–12, or 0–12 mm), and total thickness zones (0–2 or 2–6 mm) compared to unaffected eyes. Coincidently, Simsek and colleagues [14] reported a significant elevation in densitometry values of midstromal zones (2–6 or 6–10 mm), posterior zones (all), and total thickness zones (2–6 or 6–10 mm) in eyes with FUS compared to unaffected eyes. This indicated that even though the cornea observed by slit lamp is “clear” in FUS, actually the clarity of cornea is decreased. It is known that corneal clarity is maintained depending on intact corneal structure, a framework of collagen fibrils arranged in a special manner and a functional corneal endothelium [27]. Among them, the corneal endothelium plays an essential role in preserving stromal dehydration, thereby maximizing the fidelity of light passing through the cornea [27]. However, endothelial cell loss in FUS eyes have been widely reported [12, 14, 16, 18]. Our study also showed that ECD was significantly lower in affected eyes. CV and ACA, respectively, were significantly higher in FUS eyes compared to unaffected eyes. Sravani et al. also reported similar changes in these corneal endothelial parameters in Indian populations [28]. In addition, the densitometry values of center, posterior, and total layers with various diameters were confirmed as strongly related to ECD, HEX, and ACA. Simsek and colleagues [14] reported similar results. Therefore, we hypothesize that chronic inflammation in the anterior segment and ocular hypertension in subjects with secondary glaucoma resulted in numerical loss and redistribution of endothelial cells, impairing the barrier function of the endothelial layer and contributing to a net influx of aqueous fluid into the cornea presenting as greater corneal densitometry in the stromal and posterior layers. Furthermore, Labbe’ et al.[17] reported large hyper-reflective deposits corresponding to KPs on the endothelium of all FUS patients. Hashida et al. [29] also reported low/moderate reflectivity of KPs in FUS. In our study, all affected eyes presented with dispersive KPs to varying degrees (Fig. 2), which may lead to increased densitometry of posterior zones as well. Therefore, the “clear” cornea we observed in slit lamp may be not truly transparent in FUS eyes which might affect the visual quality rather than visual acuity. Especially in secondary glaucoma, the disorder of corneal clarity and endothelium needs more attention. Additionally, since cataract is an important complication of FUS, when consider the premium intraocular lens implantation, the possible impact of reduced corneal transparency on postoperative visual quality should also be considered.

The alteration of iris is a characteristic sign of FUS. Other than conventional slit-lamp biomicroscope, researchers also applied some more delicate and advanced approaches to observe and evaluate the iris change in FUS. In 1978, Melamed et al. [30] investigated the irises of two FUS patients by electron microscopy and described abnormal melanocytes with relatively few, small, and at times immature, melanin granules. However, this method is invasive and difficult to perform in vivo. Afterward, using Scheimpflug imaging, Szepessy et al. [12] noninvasively observed that the iris shape had significantly decreased its convexity in all FUS eyes, but iris cannot be quantitatively assessed. With the advent of AS-OCT, investigators who attempted to quantitatively document iris atrophic changes in vivo drew conflicting conclusions by measuring iris thickness [13, 18, 19]. Therefore, in these years, Zarei et al. [9] proposed using iris SI to quantitatively document diffuse smoothness of iris anterior surface. They pointed out that prior to the advent of OCT, when using the term “atrophy” to describe iris findings in FUS, ophthalmologists often referred to a decrease in surface features largely based on observations with slit-lamp examination. They also considered SI was more precise and objective than iris heterochromia and iris thickness, which may potentially help in correctly diagnosing dubious cases. In our study, we analyzed overall SI, temporal SI, and nasal SI in affected and unaffected eyes using AS-OCT. The results demonstrated that the SI in temporal, nasal, and overall iris was significantly greater in affected eyes, which was consistent with the result of Zarei et al. Comparing the outcomes of both studies, we found that temporal, nasal, and overall SI measured by us was numerically greater than that of Zarei et al. in either affected eyes or unaffected eyes. We speculated that the difference of ethnicity could have a possible effect on the results. It is noteworthy that the enrolled patients in their study were all Iranian while in our study were all Chinese. In addition, both investigations did not belong to large sample research, a larger sample size is needed to confirm this conjecture.

Using Visante OCT (anterior segment TD-OCT), Basarir et al. [13] found wider nasal, temporal iridocorneal angle and thinner iris in FUS eyes. They concluded that atrophy of iris and the trabecular meshwork plays a major role in widening of the angle. AS-OCT has the advantage of observing the precise structure of iris; however, neither Visante OCT nor Cirrus HD-OCT (anterior segment SD-OCT) used in our study can rapidly acquire the iridocorneal angle information in all directions due to inherent limitation. Therefore, Pentacam was simultaneously used in our study to estimate iridocorneal angle in 0 to 360° meridian through three-dimensional anterior segment reconstruction. The result demonstrated that the average iridocorneal angle in affected eyes (38.10 ± 5.41°) was larger than unaffected eyes (36.01 ± 4.77°). Szepessy et al. [12] reported similar results when comparing binocular iridocorneal angle in the unilateral FUS patient by Pentacam. Because Pentacam cannot quantitatively measure the parameters of iris (e.g., iris thickness or SI), they only subjectively compared the shape of iris and supposed that the decreased convexity of iris caused widening of iridocorneal angle. In the current study, we not only used Pentacam to evaluate average iridocorneal angle, but used AS-OCT to precisely assess the change of iris by SI. Therefore, we had vigorous evidence to confirm that iris atrophy attribute to widening of iridocorneal angle.

The first limitation of our study is the small sample size; a larger future study of patients with unilateral FUS would substantiate validity. Second, we compared subjects' affected eyes with contralateral healthy eyes; the addition of a normal healthy control group would strengthen the study design.

In conclusion, we used contemporary technology including Scheimpflug imaging camera, noncontact specular microscopy, and AS-OCT to elucidate the anterior segment disorders in Chinese patients suffering from unilateral FUS. It appears that corneal volume is a more meaningful approach to characterize true corneal thickness changes, especially in the setting of transparent cornea by slit-lamp examination after repeatedly elevated IOP. Densitometry values could provide additional evidence for corneal micro-swelling in these “clear” corneas and yield valuable information regarding corneal endothelial function along with ECD, AVA, polymegathism, and pleomorphism. In eyes without obvious heterochromia, SI and iridocorneal angle may provide additional insight for diagnosis, especially in dark or brown-colored eyes. Our findings supplement previous data of Chinese patients with unilateral FUS.

## Data Availability

The data generated during or/and analyzed during the current study are available from the corresponding author.
